# Association of prepubertal obesity with pubertal development in Chinese girls and boys: A longitudinal study

**DOI:** 10.1002/ajhb.23195

**Published:** 2018-11-02

**Authors:** Wenyan Li, Qin Liu, Xu Deng, Yiwen Chen, Bo Yang, Xin Huang, Truls Østbye

**Affiliations:** ^1^ School of Public Health and Management, Research Center for Medicine and Social Development, Collaborative Innovation Center of Social Risks Governance in Health Chongqing Medical University Chongqing China; ^2^ Department of Community and Family Medicine and Duke Global Health Institute Duke University Durham North Carolina

## Abstract

**Objectives:**

The purpose of this study was to examine the association of prepubertal body mass index (BMI) and weight status with pubertal development in boys and girls in Chongqing, China.

**Methods:**

In a longitudinal study, 1237 students (695 boys and 542 girls) were recruited from Chongqing, China, and examined at baseline, then followed every 6 months for three and a half years. Height, weight, testicular volume, and breast development were measured at every examination. Age of first spermatorrhea and menarche were obtained from self‐report. Subjects were divided into normal weight, overweight, and obese groups according to baseline BMI. Multivariable Cox regression analysis was used to examine the association of BMI and weight status with pubertal development.

**Results:**

In girls, higher prepubertal BMI increased the chance of earlier menarche (hazards ratio (HR): 1.205, 95% confidence interval (95% CI): 1.151‐2.261) and breast development (HR: 1.092, 95% CI: 1.045‐1.142). Girls in the overweight (HR: 2.605, 95% CI: 1.716‐3.956) and obese (HR: 2.565, 95% CI: 1.603‐4.103) groups had an increased risk of early menarche compared with those in the normal weight group, while only overweight was associated with an increased risk for earlier breast development (HR: 1.469, 95% CI: 1.024‐2.108). In boys, higher prepubertal BMI was significantly associated with the timing of first spermatorrhea (HR: 1.054, 95% CI: 1.004‐1.106) and testicular development (HR: 1.098, 95% CI: 1.063‐1.135). Overweight (HR: 1.672, 95% CI: 1.204‐2.322) and obesity (HR: 1.598, 95% CI: 1.135‐2.249) increased the hazard of earlier testicular development compared with the normal weight group, while no significant differences were detected among the three weight groups in terms of time to first spermatorrhea.

**Conclusions:**

Higher prepubertal BMI was associated with earlier puberty in both Chinese boys and girls.

## 1 INTRODUCTION

In 1993, after the analysis of the long‐term follow‐up data of 181 normal children, Frisch (1993) proposed the concept of “critical weight,” that is, a certain percentage of body fat is necessary for the initiation of menarche, and a certain percentage of body fat is necessary for the maintenance of fertility. There is a link between estrogen and fat storage, and this fat‐storing capacity stems from an evolutionary advantage because women need fat for conception, fetal development, and lactation. Fat also affects male fertility. Undernutrition can cause male reproductive dysfunction, and, to a certain extent, fat content is a manifestation of nutritional status. In short, a certain level of fat is a key factor in the initiation of puberty and pubertal development, but it is unclear whether obesity caused by overnutrition alters the association between fat and puberty development.

Puberty is a period during which sexual maturation occurs and reproductive capacity is attained (Golub et al., 2008; Patton & Viner, 2007). It is a milestone in the growth and development of adolescents. From the beginning of the 19th century to present, a trend toward earlier puberty has been observed globally both in girls and boys (de Muinich Keizer & Mul, 2001; Herman‐Giddens, Wang, & Koch, 2001; Ong, Ahmed, & Dunger, 2006; Parent et al., 2003; Tinggaard et al., 2012).

In view of the increasing incidence of overweight and obesity among children during the same period (Chinn & Rona, 1994; Ji & Chen, 2013; Mindell, Dinsdale, Ridler, & Rutter, 2012; Wang & Lim, 2012), the study of weight status and early pubertal development has attracted attention. There have been numerous studies of the relationship between body fat and pubertal development, and whether there is a relationship or interaction between them is still the focus of debate. In girls, there have been numerous longitudinal studies demonstrating an inverse association between obesity and puberty timing, as defined by age of menarche, breast development, and pubic hair development (Gavela‐Pérez, Garcés, Navarro‐Sánchez, López; Villanueva, & Soriano‐Guillén, 2015; Ramezani Tehrani, Mirmiran, Gholami, Moslehi, & Azizi, 2014; Sloboda, Hart, Doherty, Pennell, & Hickey, 2007; Zhai et al., 2015), while a few studies have not found this association (Ferrández et al., 2009; Mouritsen et al., 2012). In boys, there have been fewer studies, and the results have been more ambiguous. Wang (2002), Kleber, Schwarz, and Reinehr (2011) and Lee et al. (2010) reported a negative correlation between body fat and pubertal development in boys, Vizmanos and Martí‐Henneberg (2000) found the opposite, and Mouritsen et al. (2012) failed to find any associations. A recent systematic review and meta‐analysis (Li et al., 2017) of obesity and pubertal development noted that obesity might contribute to early onset of puberty in girls, while in boys there is insufficient data. Meanwhile, there have been few longitudinal studies among Asians, most studies to date have focusing on European and Black populations.

While there appears to be a relationship between body weight and pubertal development in both girls and boys, the nature of this relationship needs to be further studied. As the current relevant research has been mostly cross‐sectional, larger, longitudinal studies are needed to validate the association.

We, therefore, conducted a three and a half year prospective cohort study to further investigate, the association of overweight and obesity with pubertal development among Chinese primary school girls and boys who had not yet started puberty at the beginning of the study.

## 2 METHODS

The study subjects were selected from an ongoing cohort study of grades one to four primary students from four elementary schools in Chongqing, southwest China. This cohort study was designed to track the onset and process of pubertal development and assess the factors influencing pubertal development in both girls and boys. A total of 1237 children were recruited after written consent had been obtained both from the students themselves and from their guardians before the baseline survey conducted in May, 2014. As of December 2017, seven follow‐up surveys (every 6 months) had been completed. Physical examination, including pubertal development stages, height and weight measurements were conducted by trained medical school graduates during each survey.

This study was approved by the Medical Ethics Review Committee of Chongqing Medical University.

### 2.1 BMI measurement

During each anthropometric examination, weight and height were measured by trained medical school graduates using standard procedures. Children were asked to take off their shoes and remove their hair clips before the measurement of their height and weight. Standing height was measured to the nearest 0.1 cm using a stadiometer, weight was measured to nearest 0.1 kg using a balance scale. Height and weight were used to calculate BMI (kg/m^2^). BMI cut‐offs for overweight and obesity were in accord with the BMI reference norms for screening overweight and obesity in Chinese children and adolescents from the Group of China Obesity Task Force (Ji, 2004), based on more than 244 200 students aged 7 through 18 years.

### 2.2 Pubertal development

Pubertal development (breast development in girls and testicular development in boys) was assessed according to Tanner stages (Tanner, 1986) by trained medical school graduates during physical examination. Investigators guided the girls and boys to recall whether their menarche or first spermatorrhea had occurred, and, if so, asked them to recall the exact time of the occurrence to the nearest month. Onset of puberty was defined in the following two ways in girls: (1) had experienced menarche according to self‐report; (2) breast development had reached stage 2 by the time of the examination. In boys, the beginning of pubertal development was also defined in two ways: (1) had experienced first spermatorrhea according to self‐report; (2) testicular volume had reached 4 mL in physical examination.

### 2.3 Statistical analysis

Data were entered using Epidata 3.0 (Jens M. Lauritsen, Michael Bruus, and MarkMyatt, Odense, Denmark) and double‐entry verification was carried out. Descriptive statistics were presented as means and standard deviations for continuous variables and as percentages for categorical variables. Differences in mean age across the different weight status groups were assessed using Kruskal‐Wallis tests. Given that not all children had begun their pubertal development at the last follow up survey, we used Cox regression analysis to analyze the effects of overweight and obesity status on pubertal development; the outcome is the time until the event. Separate analyses were conducted for each of the four different outcome measures of puberty. BMI at baseline was used as the independent variable to first perform unadjusted, univariate Cox regression analysis. With BMI as the independent variable and age at baseline as the control variable, multivariable Cox regression analysis was then performed. We defined observation time as the time from when the children entered into the cohort (baseline) until start of pubertal development. If pubertal development had not started at the time of the last follow‐up, observation time was calculated from baseline until the last follow‐up (censored observations).

Testicular development and breast development were assessed by the investigators at each time of follow‐up; however, the onset of testicular development and initiation of breast development were assumed to have occurred at a time point between the two follow‐ups. Therefore, the midpoint of the follow‐up period between pubertal development initiation and the previous time point was set as the initiation time for testicular development and breast development. There were several cases of censored data in our analysis: (1) children who had not yet started pubertal development at the last follow‐up, and (2) children who were lost to follow‐up for various reasons during the study period. SPSS 21.0 (Version 21.0 for Windows, Chicago, IL) was used for data analysis. Significance was set at the .05 level in all analyses.

## 3 RESULTS

### 3.1 Characteristics of participants

**Figure 1:**
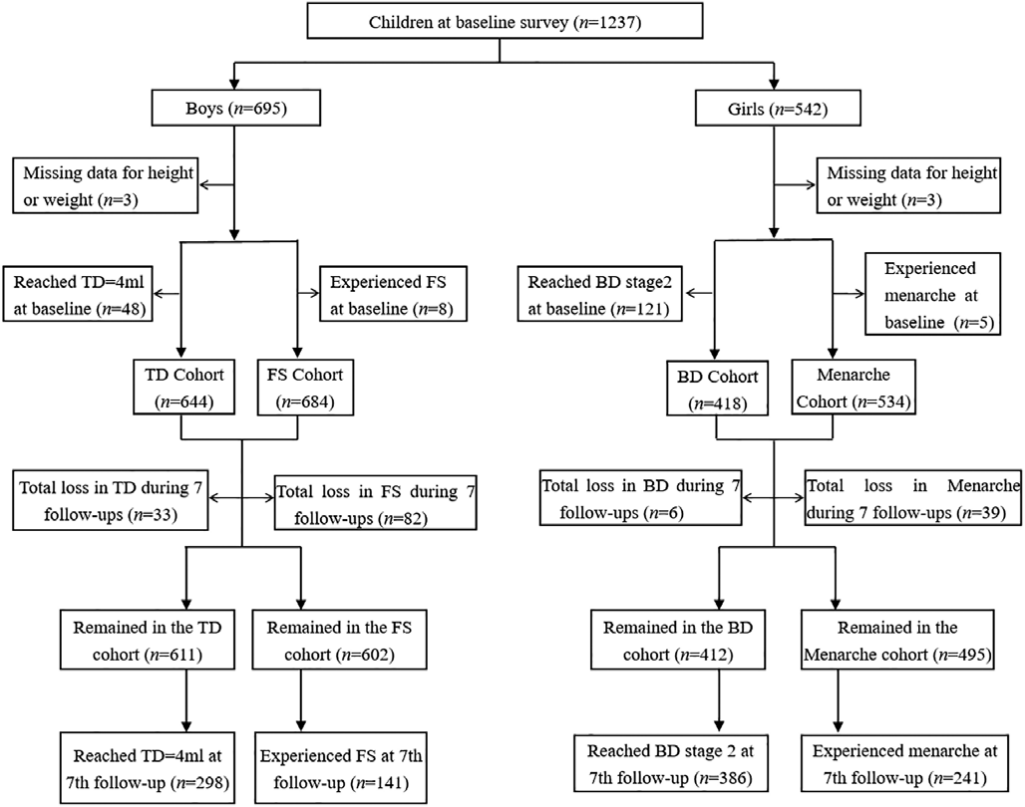
Longitudinal observation flowchart relating to four pubertal development outcome measures, follow up over three and a half years. *TD = testicular development; FS = first spermatorrhea; BD = breast development

A total of 1237 children were enrolled in the baseline survey, 695 boys and 542 girls, mean age was 8.59 (SD:1.20) with a range of 5.81‐12.20 years. The prevalences of overweight and obesity were 8.9% and 6.8% in girls and 11.5% and 10.2% in boys at baseline. Those with missing data for height and/or weight and those who experienced pubertal development at baseline for each indicator were excluded from the analyses. Four outcome measures were established: menarche, first spermatorrhea, testicular development, and breast development. Normal weight, overweight, and obese status were based on BMI at baseline, and the number of children in each weight status group for the four outcomes at follow‐up surveys are shown in Figure 1.

The loss to follow up rate was 7.30%, 1.44%, 11.99%, and 5.12% for the menarche, breast development, first spermatorrhea, and testicular development analyses, respectively. The main reasons for the loss to follow up included transfer to another school, entry into a higher school, or refusal to participate in the investigation. There were no significant differences in the age, BMI or distribution of weight status among subjects lost to follow‐up, except for those lost in the first spermatorrhea analysis, who were significantly older than those in the cohort.

### 3.2 Age of each group

As shown in Table 1, the Kruskal‐Wallis test showed that mean age was statistically different between different weight status groups in the analysis of breast development and first spermatorrhea (*P* < .05).

**Table 1:** Comparison of age between different weight groups for four outcomes

Outcome	Weight group	Age, mean (*SD*), y	χ^2#^	*P*
Menarche	NW	8.62 (1.21)	1.48	.228
	OW	8.33 (1.13)		
	OB	8.46 (1.23)		
Breast development	NW	8.28 (1.06)	3.86	.022
	OW	7.90 (0.88)		
	OB	7.83 (0.93)		
First spermatorrhea	NW	8.52 (1.20)	3.13	.044
	OW	8.87 (1.10)		
	OB	8.52 (1.04)		
Testicular development	NW	8.44 (1.17)	2.19	.112
	OW	8.74 (1.08)		
	OB	8.50 (1.03)		

#: Kruskal‐Wallis test to determine whether there was a significant difference in age between groups.

Abbreviations: NW = normal weight group, OW = overweight group, OB = obese group.

### 3.3 BMI, weight status, and pubertal development

**Figure 2:**
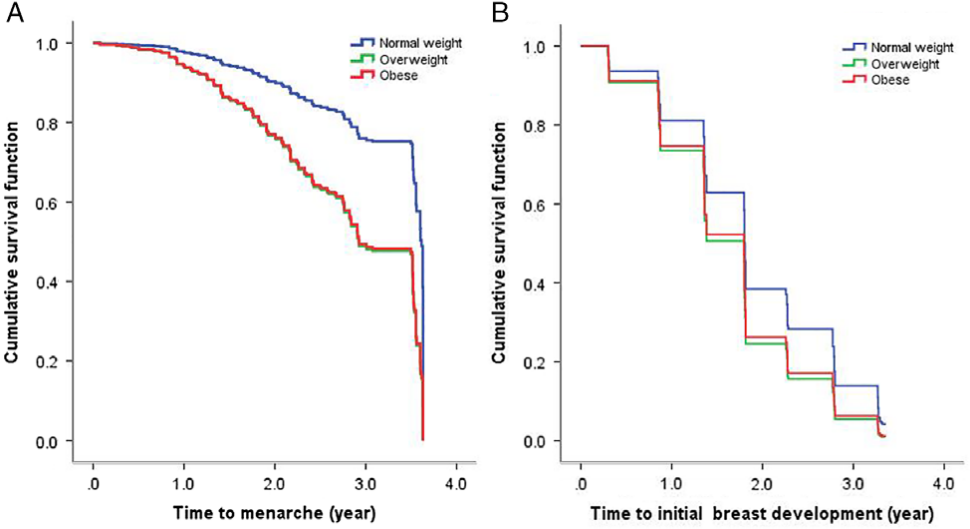
Survival curves with menarche and breast development as end points in normal weight, overweight and obese girls, adjusted for age

Multivariable Cox regression analysis of 534 girls with menarche as the outcome showed that one‐unit (kg/m^2^) increase in BMI was associated with a 20.5% (95% CI: 15.1%‐26.1%) increased risk of experiencing menarche, adjusting for age. In analyses stratified by baseline BMI categories, compared to the normal weight group, overweight girls had a significantly higher risk of experiencing menarche with HR = 2.605 (95% CI: 1.716‐3.956), as had obese girls with HR = 2.565 (95% CI: 1.603‐4.103). See Table 2 for details and Figure 2A for the survival curves with menarche as the end point for the three weight status groups.

**Table 2:** Association between prepubertal BMI and pubertal development in children: Results from multivariate cox regression

Variable	Menarche (*n* = 534)		Breast development (*n* = 418)		First spermatorrhea (*n* = 684)		Testicular development (*n* = 644)	
	HR^a^ (95% CI)	*P*	HR (95% CI)	*P*	HR (95% CI)	*P*	HR (95% CI)	*P*
Continuous variable								
BMI (kg/m^2^)	1.205 (1.151‐1.261)	<.001	1.092 (1.045‐1.142)	<.001	1.054 (1.004‐1.106)	.033	1.098 (1.063‐1.135)	<.001
Age (years)	2.545 (2.259‐2.868)	<.001	1.726 (1.565‐1.903)	<.001	3.103 (2.586‐3.724)	<.001	2.681 (2.390‐3.007)	<.001
Categorical variable								
Normal weight^b^	–	<.001	–	.046	–	.468	–	.001
Overweight	2.605 (1.716‐3.956)	<.001	1.469 (1.024‐2.108)	.037	1.297 (0.818‐2.056)	.270	1.672 (1.204‐2.322)	.002
Obese	2.565 (1.603–4.103)	<.001	1.399 (0.913‐2.145	.123	1.210 (0.714‐2.051)	.479	1.598 (1.135‐2.249)	.008

a HR for BMI and different weight groups was adjusted for age.

b Normal weight is the reference group.

A total of 418 girls were included in the survival analysis for breast development, of whom 386 (92.3%) had experienced breast development up to the seventh follow‐up. Multivariate Cox regression analysis showed that BMI was significantly related to breast development with an HR of 1.092 (95% CI: 1.045‐1.142). After categorization into BMI groups, only overweight (HR: 1.469, 95% CI: 1.024‐2.108) girls had earlier breast development compared with normal weight girls. Table 2 shows the hazard ratio with 95% CI for the different groups. Figure 2B shows the survival curves with breast development as the end point, comparing the three weight status groups.

**Figure 3:**
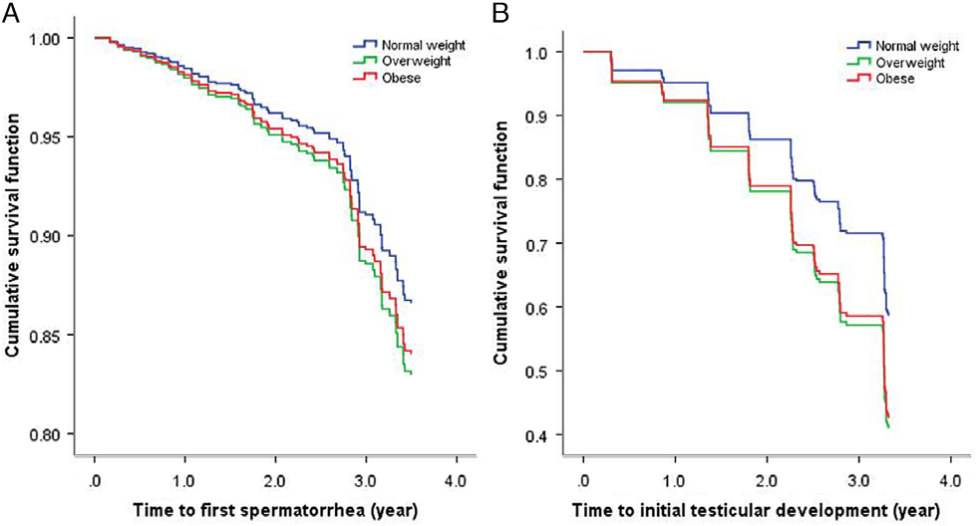
Survival curves for first spermatorrhea and testicular development as end points comparing normal weight, overweight and obese groups, adjusted for age

A total of 684 boys were included in the survival analysis with first spermatorrhea as the end point. As of the seventh follow‐up, 141 boys (20.6%) had experienced their first spermatorrhea. Multivariate Cox analysis showed that BMI (HR: 1.054, 95% CI: 1.004‐1.106) was associated with first spermatorrhea. Boys with higher BMI experienced first spermatorrhea earlier. There were no statistically significant differences in time to first spermatorrhea between the overweight, obese, and normal weight groups (Table 2, Figure 3A).

Multivariable Cox regression analysis of 644 boys showed that higher BMI was also associated with earlier testicular development (HR: 1.098, 95% CI: 1.063‐1.135). In analysis by BMI group, overweight (HR: 1.672, 95% CI: 1.204‐2.322) and obese (HR: 1.598, 95% CI: 1.135‐2.249) boys had earlier testicular development compared with normal weight boys. See Table 2 and Figure 3B for details.

## 4 DISCUSSION

To our knowledge, this is the first longitudinal study of a relatively large number of both Chinese girls and boys to examine the association between prepubertal BMI and pubertal development. The study included different outcome measures related to pubertal development and the subjects were followed for three and a half years with follow up assessments of puberty status every 6 months. The results suggest that higher prepubertal BMI was significantly associated with earlier menarche and breast development in girls, and with earlier first spermatorrhea and testicular development in boys. Furthermore, when subjects were divided into overweight and obesity groups, overweight and obese children experienced earlier menarche and testicular development, and overweight girls experienced earlier breast development compared with normal weight girls. However, overweight and obese boys were not statistically different from normal weight boys in terms of time to first spermatorrhea.

Age of menarche has been widely used in previous studies for its specificity and ease of collection, but, if used, younger girls could not be studied. This study included both breast development and menarche as outcome measures for puberty development in girls, so that prepubertal girls of young ages could also be included. Several studies (Gavela‐Pérez et al., 2015; Ramezani Tehrani et al., 2014; Sloboda et al., 2007; Zhai et al., 2015) have shown that overweight and obesity status is positively correlated with early puberty. Davison, Susman, and Birch (2003) conducted a longitudinal study with a sample of 183 white girls, and demonstrated that girls with higher percent body fat at 5 and 7 years were more likely to experience early pubertal development at 9 years. Another cohort study (De Leonibus et al., 2014) with 84 prepubertal girls also showed that obese girls entered puberty earlier. However, these studies had small sample sizes and their breast development examination only used the inspection method. We used both inspection and palpation to assess the breast development in girls to avoid overestimation of breast development in obese girls. In addition, at every physical examination, we kept the last measurement data record for reference to minimize judgment errors.

It took about 2.6 years for 40% of the girls in the overweight and obese group to experience menarche, that is, 1 year earlier than that for normal weight girls. After 3.5 years of observation, about 56.3% of the girls in the overweight and 54.1% in the obese group had reached menarche compared with 43.2% in the normal weight group. For breast development, it took about 1.8 years for 70% of the overweight and obese girls to reach breast development stage 2, which was about 1 year earlier compared with normal weight girls. After 2 years, about 74.3% and 70.8% of the girls in the overweight group and obese group had stage 2, respectively, whereas in the normal weight group, 58.5% had reached stage 2. However, there was no statistically significant difference in time to breast development between obese and normal weight girls. This may be because the number of obese girls in our study was relatively small. Furthermore, the results also suggest that the impact of body fat on pubertal development might not be linear, and overweight and obese girls may differ in their pubertal development.

There are far fewer studies on the relationship between obesity and pubertal development in boys. This is likely because the early puberty in girls attracted public attention earlier. Furthermore, studies of boys rely on physical examination to record the accurate stage of penis or testicular volume, and such examination can be more difficult and time consuming in large‐scale cohort studies.

The first spermatorrhea, which indicates male sexual maturity, is commonly used as the indicator of the late stage of puberty in boys. The number of previous studies with first spermatorrhea as an outcome measure is limited. The reason may be that it takes longer to observe the occurrence of the first spermatorrhea than other indicators in prospective studies, and, in retrospective studies, recall bias is unavoidable. In our study, BMI was significant when analyzed as a continuous variable but not when grouped into weight categories. This may be due to information lost when converting a continuous into a categorical variable. Further, the prevalences of overweight and obesity status among boys in our study were relative low, 11.5% and 10.2%, respectively, resulting in small sample size for detecting differences. After about 3.5 years of observation, about 19.3% of the boys in the normal weight group experienced first spermatorrhea, while the proportions in overweight and obese groups increased by 8.2% and 3.2%, respectively. Consistent with these findings, the results of the first nationwide cross‐sectional study of boys' pubertal development in China showed that age at first spermatorrhea was earlier for boys with BMI above the 85 percentile (Sun et al., 2012). Moreover, data from 63 625 boys included in the China 2010 National Physical Fitness and Health Surveillance (Wen, Zhu, & Wang, 2015), using the same criteria as our study to define obesity, also showed that age of first spermatorrhea in the obese group was 0.1 years earlier than in the nonobese group. However, these studies were limited by their cross‐sectional design.

According to the Tanner standard (Tanner, 1986), the development of genitalia can be divided into five periods (G1 to G5), with G2 as the marker of puberty initiation. External genital development includes penile and testicular development, and most studies have used testicular volume as the indicator of puberty. Our study used testicular volume equal to 4 mL as the initiation of puberty according to the Tanner standard, and only those whose testicular volumes were below 4 mL at the baseline were included in the cohort. The higher the prepubertal BMI of the boys in the same age group, the earlier the age of puberty, and this finding was also significant after dividing subjects into overweight and obese groups. Our survival curve also illustrates that it took around 1.8 years and 2.3 years for 30% of boys in the overweight and obese groups to reach testicular development stage 2, respectively, while it happened 0.8 years later in the normal weight group compared with the obese group. After 3.5 years of observation, about 59.7% of overweight boys and 56.5% of obese boys reached stage 2 compared with about 42.9% of the normal weight group. Consistent with our findings, a prospective longitudinal study conducted from 2005 to 2012, among 71 prepubertal European boys showed that the mean age of boys reaching 4 mL of testicular volume was younger in the obese group than in the normal‐weight group (De Leonibus et al., 2014). Similar results were also found in Danish (Sørensen, Aksglaede, Petersen, & Juul, 2010) and Chinese study (Liang, Du, Gu, et al., 2004). Contrary to the above results, a Spanish longitudinal cohort study showed a positive relationship between BMI and the age of pubertal onset (Vizmanos & Martí‐Henneberg, 2000). In addition, in the United States, Lee et al. (2010) conducted a 10‐year prospective study of 401 U.S. boys born in 1991, and showed that the number of boys reaching Tanner stage 2 genitalia in the lowest BMI trajectory group was higher than in the highest BMI group at age 11.5 years. However, BMI for the 304 noneligible boys who were excluded from the study was higher than that of the included boys.

The results of studies to date of the effects of overweight and obesity on testicular development in boys are not consistent; the possible reasons may be as follows. First, subjects in these studies vary in race/ethnicity, and there are differences in the occurrence of overweight and obesity status, and in pubertal development for different race/ethnicities. Wang (2002) has suggested that racial/ethnic differences in obesity are related to racial/ethnic differences in sexual maturity. Second, children in different social and cultural groups have different eating habits and nutritional status, which may also affect growth and pubertal development. Third, the criteria used to determine obesity in each study were different, or the same puberty indicator was used but there were different criteria for determining the categories of early or late. Finally, it is noteworthy that BMI as a single indicator of body fat may not fully reflect obesity. Increase in the BMI levels of boys during adolescence appears to be mainly due to an increase in fat‐free mass rather than body fatness. Therefore, there is not necessarily an association between the increase of BMI and the increase of body fat in boys during puberty. The Spain cohort showed a positive relation between age of pubertal onset and BMI, but no significant differences were found between subcutaneous skinfolds, upper arm fat estimate and percentage of body fat mass (Vizmanos & Martí‐Henneberg, 2000). Therefore, besides BMI, body fat measurements, including the use of skinfold thickness and bioelectrical impedance, should also be considered when measuring body fat in future studies.

The mechanism for the association between obesity and puberty is not fully understood. Several potential hormones and mechanisms that may explain the association between obesity and pubertal timing have been described. Estrogen, a major class of female hormones, promotes the maturity of female sexual organs and the emergence of secondary sexual characteristics, and fat serves as the primary extragonadal source of estrogen in girls. In addition, the mechanisms of obesity promoting gonadal axis initiation include leptin. Leptin levels are positively correlated with obesity, which may be associated with leptin resistance, while leptin also stimulates the central pulsatile gonadotropin secretion and may trigger the timing of puberty by binding to receptors in the GnRH neurons (Ahima, Dushay, Flier, Prabakaran, & Flier, 1997; Chan et al., 2006; Fu & Zhou, 2014). Leptin‐deficient mice and humans fail to enter puberty unless leptin is administered, but an injection of leptin for mice or children with leptin deficiency alone cannot induce puberty (Cheung et al., 1997; Farooqi, 2002). This shows that leptin as a marker of adipose tissue may be an important permissive factor for puberty initiation, rather than the critical factor in puberty. In addition, due to the increasing secretion of growth hormone and insulin‐like growth factor (IGFI), insulin sensitivity decreases and compensatory hyperinsulinemia occurs which is exacerbated by obesity, especially female obesity. Hyperinsulinemia increases estrogen levels, which promotes breast development (Ahmed, Ong, & Dunger, 2009; Holly et al., 1989; Shimizu, Oh‐I, Okada, & Mori, 2007). Moreover, obesity is often accompanied by inflammatory reactions that increase cytokines and promote the synthesis of androgen; such changes in androgen may precipitate early pubertal development (Blank et al., 2009). These observations may explain some of the effect of body fat on puberty that we observed in this study.

Even though we have conducted a rigorous longitudinal study with regular repeated assessment of physical growth and pubertal development for prepubertal children, some limitations still need to be acknowledged. First, our study was conducted in Chongqing, China, and all the participants were Chinese, therefore differences between race/ethnicities should be taken into account when extrapolating our findings. Second, BMI was used as the main indicator in our study to determine overweight and obesity in children, which may not fully reflect body fat level as BMI only considers height and weight in the calculation. Further studies are recommended using skinfold thickness or other measurements such as dual‐energy x‐ray absorptiometry to further examine the association between body fat and puberty timing. Third, we considered only the prepubertal BMI of subjects, which can explain the influence of prepubertal BMI and weight status on pubertal development, but cannot clarify the effects of weight changes during follow up on pubertal development. Fourth, some subjects in the cohort had not entered puberty yet at the last follow up, reducing the power of the study.

## 5 CONCLUSION

This longitudinal study provides evidence that higher prepubertal BMI is associated with earlier pubertal development in both girls and boys in China. Overweight and obese girls experience menarche earlier, and overweight girls are more likely to have earlier breast development. Overweight and obese boys also tend to start testicular development earlier.

## CONFLICT OF INTEREST

The authors report no conflicts of interests.

## AUTHORS CONTRIBUTIONS

QL and WL participated in the design of the study. QL, WL, XD, YC, BY, and XH were involved in data collection. QL, WL, and TO designed the analysis strategy and interpreted the results. QL and WL analyzed the data. WL prepared the first draft. QL and TO revised the manuscript. All authors read and approved the final manuscript.
